# Type 2 Diabetes Mellitus in Patients With Polycystic Ovary Syndrome

**DOI:** 10.7759/cureus.46859

**Published:** 2023-10-11

**Authors:** Anjali Agrawal, Apoorva Dave, Arpita Jaiswal

**Affiliations:** 1 Obstetrics and Gynecology, Jawaharlal Nehru Medical College, Datta Meghe Institute of Higher Education and Research, Wardha, IND

**Keywords:** insulin resistance, glucose, endocrine, pcos, diabetes mellitus

## Abstract

Polycystic ovary syndrome (PCOS) is a multisystemic disorder usually seen in females who are in their reproductive age (15-49 years of age). PCOS exhibits insulin resistance and hyperinsulinemia, which make it a pre-diabetic state. The syndrome has many overt changes, like dyslipidemia and hypertension, which increase the risk of cardiovascular diseases. There is also an increased risk of development of hepatic steatosis. Resistance to insulin, increased amount of insulin, and dysfunction of beta-cells are frequent in PCOS, although they are not the only cause for diagnosis. Type 2 diabetes and glucose resistance may result from total or compared insulin insufficiency, which can happen if the beta cells' compensatory response slows down. Pregnancy challenges such as miscarriage, gestational diabetes mellitus (DM), hypertensive disorders of pregnancy, more excellent rates of cesarean birth, and abnormalities in fetal development may be more common in women with PCOS. In studies investigating the glucose-insulin system compared to control groups with similar age and weight, glycemic intolerance, which includes both decreased glucose tolerance and type 2 diabetes, was more common in PCOS women. In the short-term therapy of insulin resistance in PCOS, the potential use of insulin-sensitizing medications has recently been studied. Controlled studies have demonstrated that metformin treatment can lower fasting and stimulate plasma insulin levels by encouraging body weight reduction. These findings provide insulin-sensitizing drugs as a unique method in treating ovarian hyperandrogenism and irregular ovulation in PCOS and indicate a new prescription for Metformin. They further assert that long-term metformin treatment may assist in addressing insulin resistance, reducing the risk of type 2 diabetes and cardiovascular-related disease in people who take it.

## Introduction and background

Polycystic ovary syndrome (PCOS) is a pervasive disorder involving the endocrine system among women who are of reproductive age. The traditional signs of PCOS include polycystic ovarian morphology, hyperandrogenism, and ovarian dysfunction (menstrual irregularity or subfertility) [1]. The intrauterine environment, which affects fetal development, is mostly responsible for PCOS in 80% of cases [2]. It is also inherited with resistance to insulin since childhood. PCOS can also arise as a result of hyperandrogenism throughout puberty. Insulin resistance is often seen in women with PCOS due to hormonal imbalances. This resistance is more marked in obese women, which shows that obesity and PCOS have a symbiotic relationship concerning the magnitude of insulin. Obesity, elevated testosterone, and low levels of sex hormone-binding globulin are additional characteristics of PCOS [3]. PCOS is a multisystemic disorder and can also involve the cardiovascular system. Diabetes mellitus (DM) is caused by the body's resistance to insulin or a deficiency of insulin synthesis from the pancreatic islet cells of Langerhans. The classical features of DM include polyphagia, polydipsia, and polyuria. The precise chance of developing DM in people with PCOS is unknown. Insulin resistance, a characteristic of DM and PCOS, can eventually result in DM, nevertheless.

## Review

Methodology

**Figure 1:**
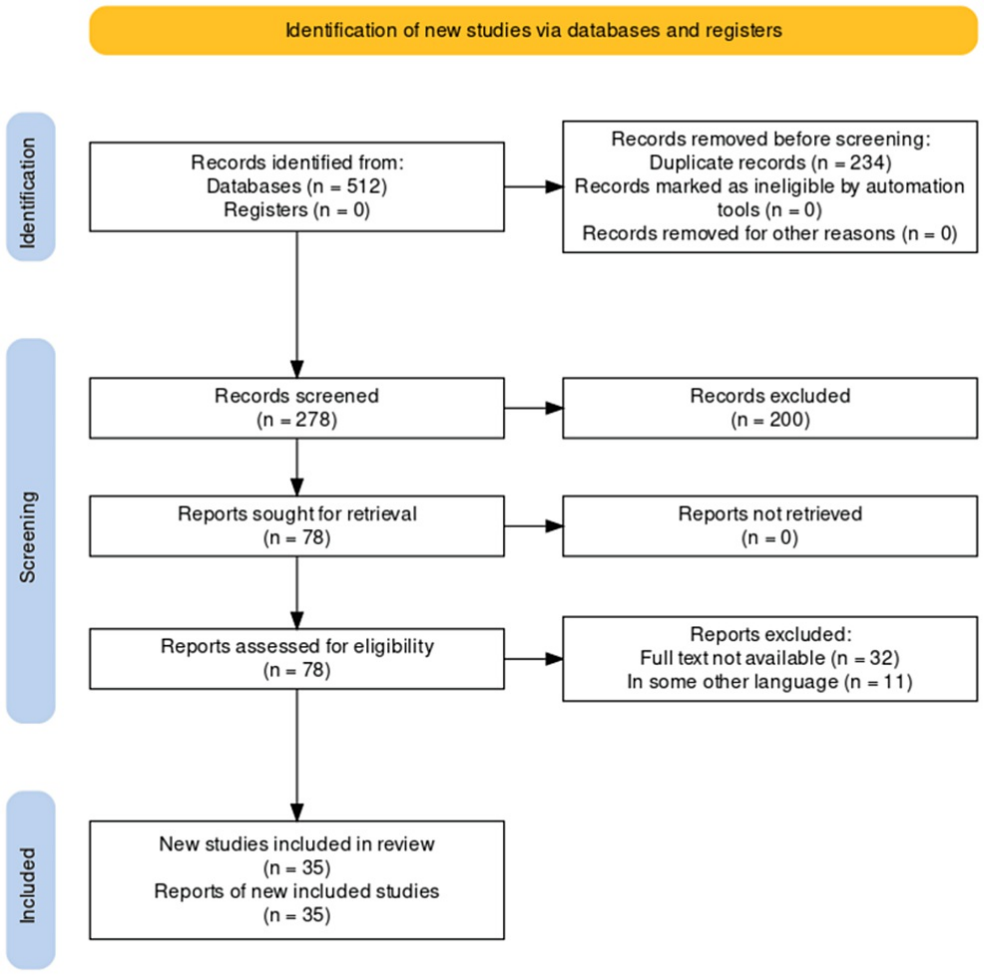
PRISMA Flow Diagram for Diabetes Mellitus and Polycystic Ovarian Syndrome Credit: Author PRISMA, Preferred Reporting Items for Systematic Reviews and Meta-Analyses

The relationship between DM and PCOS was thoroughly reviewed using a literature search. Using the following keywords and combinations: insulin resistance, glucose, endocrine, PCOS, DM, and more, we searched several electronic databases, including PubMed, MEDLINE, Embase, and Google Scholar. Articles published between 2000 and 2023 were included in the search. Reference lists of pertinent publications and review papers were manually examined in addition to electronic database searches to find more studies. The selection process for the research that satisfied the inclusion criteria included observational studies, experimental studies, systematic reviews, and meta-analyses that looked at the relationship between DM and PCOS, and how they affected related outcomes. The inclusion of only peer-reviewed, published articles was taken into consideration. Titles, abstracts, and full-text publications were evaluated independently by two reviewers, and any inconsistencies were settled by discussion and agreement. The extensive literature search provided a complete examination of the relationship between DM and PCOS, which aimed to assure the inclusion of pertinent research. The method utilized to choose the papers for our study is shown in Figure 1.

Polycystic ovary syndrome

**Figure 2:**
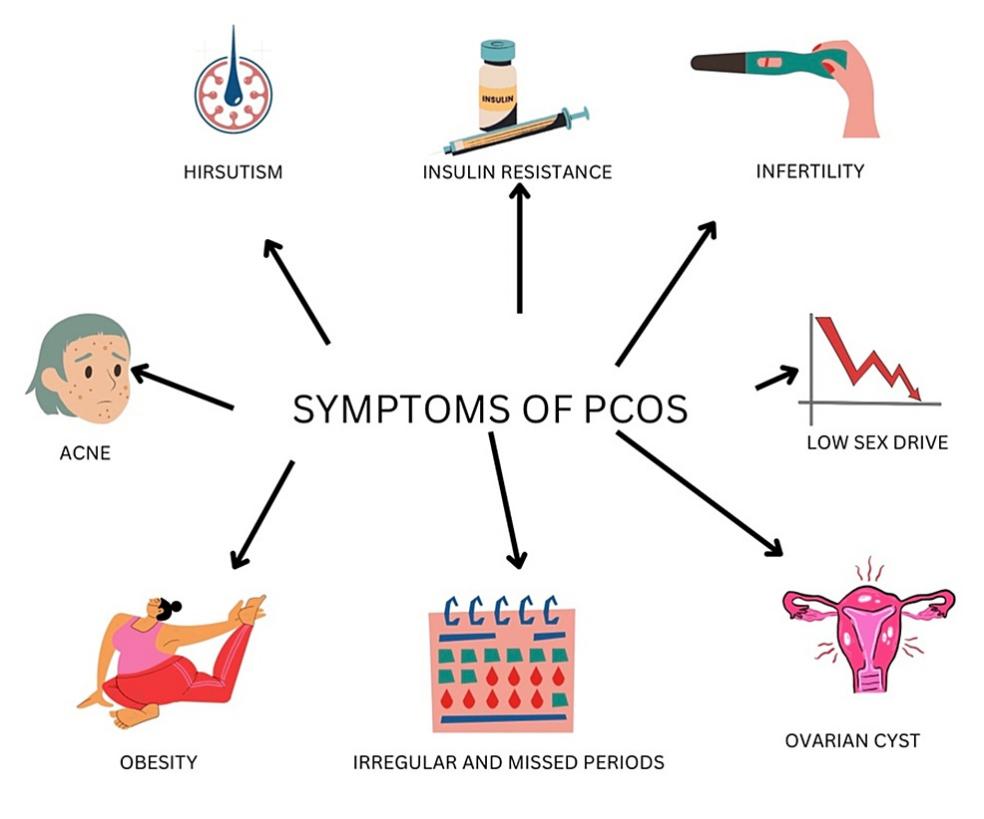
Common Clinical Signs and Symptoms of PCOS Credit: Author PCOS, polycystic ovary syndrome

Stein-Leventhal syndrome is another term for PCOS. It is defined as chronic anovulation and hyperandrogenism, which occur in women of reproductive age [4]. Women in the reproductive age range are 10% more likely to get the syndrome. There are three main components of PCOS: increased androgens (hyperandrogenism), ovulatory dysfunction, and cysts (unruptured follicles) in ovaries. The risk factors for developing PCOS include obese women, a history of premature adrenarche, and a history of first-degree relatives with PCOS [3]. PCOS shows genetic inheritance and runs in families. A bimodal distribution of testosterone and dehydroepiandrosterone (DHEA) concentrations is observed [5]. The primary etiology behind it is the increased production of androgens by ovaries. Normal androgen levels are <70 ng/dL, but in PCOS, androgen levels are >200 ng/dL. The elevated levels of androgen show a folliculotoxic effect; hence, multiple follicles undergo arrested growth. Since there is no dominant follicle left, it will lead to anovulation. There is no corpus luteum formation. Thus, levels of progesterone remain low and lead to amenorrhea or oligomenorrhea. Due to progesterone levels, endometrial support is lost; hence, there is menometrorrhagia in obese women. Additionally, due to a lack of endometrial backing, there is a substantial chance of abortion if the woman becomes pregnant [6]. Therefore, mild elevation of androgens can cause hirsutism in females. Hirsutism is the development of coarse terminal hair with a masculine pattern over lips, chin, periareolar region, chest, and around linea nigra, acne non-responsive to treatment, and alopecia. PCOS is the most typical cause of hirsutism in young girls [7]. The typical clinical signs and symptoms are shown in Figure 2.

Type 2 diabetes was found in 32% of perimenopausal women who had previously undergone a wedge resection for polycystic ovarian syndrome [8]. The following are essential considerations for physicians highlighted by this review: alternative diagnoses must be ruled out because PCOS is a clinical diagnosis. Because PCOS contains a hereditary risk of insulin resistance independent of body mass index or grade of obesity, women with PCOS should also be evaluated for metabolic effects. Ultimately, since PCOS and infertility are related, this issue should be covered early on in women's management of PCOS [9]. PCOS is often diagnosed using the Rotterdam criteria [10]. Diagnosing PCOS patients can be done by clinical history, physical examination, hormonal testing to confirm hyperandrogenism, and oligo-anovulation. The primary goals of the treatment include suppressing and counteracting androgen secretion and action, improving metabolic status, and, finally, improving fertility [11]. Women with polycystic ovarian syndrome should be carefully evaluated, especially for developing DM of type 2 variety and gestational diabetes. When pregnancy is found, oral glucose loading should occur between weeks 20 and 32 of gestation [8].

The usual biochemical results in women with PCOS include high blood concentrations of testosterone and, less commonly, elevated levels of luteinizing hormone (LH) but normal levels of follicle-stimulating hormone (FSH). Importantly, PCOS is also linked with metabolic dysfunction, the most prominent of which is insulin resistance, and this profile has consequences for long-term health. In other words, PCOS is a metabolic condition and a reproductive problem. Because the effects of metabolic dysfunction prevail in many instances, it has been advocated that the syndrome's nomenclature be changed [12]. It is crucial to test for and treat the comorbid disorders frequently linked to PCOS, such as T2DM, obesity, NAFLD, hyperlipidemia, OSA, and anxiety, in addition to addressing the symptoms of PCOS [13]. The targeted clinical results of PCOS patients ultimately guide the patient's care, which can take many different forms [13]. Lifestyle change is the first step in PCOS management. Long-term lifestyle modifications have been shown to reduce glucose intolerance and delay complications [14]. It is essential to make lifestyle adjustments, such as switching to a more balanced diet and exercising frequently. Various treatments, including metformin, thiazolidinediones, and others, look particularly promising in treating cardiometabolic aspects of PCOS [13]. Due to anovulation, infertility is easily reversible by medical treatment with drugs such as letrozole and clomiphene citrate. Valproic acid, an antiepileptic medication, is also thought to increase the chance of developing PCOS [15].

Diabetes mellitus type 2

DM is a common endocrinopathy. Previously, it was referred to as adult-onset diabetes or non-insulin-dependent diabetes. It involves abnormal glucose, lipid, and protein metabolism, insulin resistance, and decreased insulin production [16]. The leading causes are insulin resistance and an insulin deficit brought on by the destruction of beta cells. The risk factors include genetic and environmental factors. Type 2 DM has a more aggressive effect on young people than the adult population. It is usually seen in individuals over 25, but nowadays more often in children and younger folks. It remains asymptomatic for a long time or may be subclinical. The prevalence of DM runs deep in the patients' families [17]. The significant risk factors that might lead to the development of diabetes include more prevalence in females, obesity, acanthosis nigricans, and ketoacidosis. Insulin resistance increases as the body weight in obese women increases; thus, insulin resistance is directly proportional to obesity [18]. Insulin resistance in various insulin-target organs, including the liver, muscle, and adipocytes, and pancreatic cell failure are frequent characteristics of type 2 diabetes. Normal cells may overcome insulin resistance by producing more insulin or by having more cells, but insufficient compensation leads to glucose intolerance. When hyperglycemia is identified, insulin resistance and cell function decline. The process is known as "glucose toxicity" [19].

Depending on when diabetes was first identified, during puberty, reproductive issues might arise. Women who have type 1 diabetes have traditionally experienced amenorrhea in addition to sterility because of central hypogonadism. These issues have been reduced but not eliminated due to improved metabolic management and higher insulin treatment [20]. Type 2 diabetes is a common and overlooked disease that provides treatment challenges for family doctors. Within the last three years, the development of novel oral medications has increased the variety of viable treatment combinations. Clinicians must emphasize nonpharmacologic measures such as diet modification, weight control, and regular exercise, regardless of the drug therapy [21]. Drugs to be used must be determined by patient characteristics, glucose control level, and economic concerns. Combinations of several oral medications may be effective in managing hyperglycemia before insulin treatment is required. A stepped-care strategy to medication therapy may be the most reasonable and cost-effective way to manage this condition. Clinical trial pharmacoeconomic studies are needed to find cost-effective treatment methods for DM [16].

Individual patient education and consultation improve metabolic control and should begin when type 2 diabetes is diagnosed. Limiting calories and strenuous activity, particularly in the short term, are non-pharmacological treatments for type 2 diabetes that stimulate biological mechanisms that defend the organism. The lifestyle modifications include exercise for at least 30 minutes a day, salt and sugar restriction, 30g/day dietary fiber, and moderate intake of alcohol. The quantity of fat, proteins, and carbohydrates should be adjusted according to different individuals [10]. Medical treatment aims to prevent long-term complications such as microangiopathy (retinopathy, nephropathy, neuropathy) and macroangiopathy (myocardial infarction, gangrene, diabetic foot). A single antihyperglycemic medication (monotherapy) is frequently sufficient at first, but another agent with a different type of action is typically required later on (combination treatment) [22]. When glucose levels are low, DPP-4 inhibitors, incretin mimetics, metformin, acarbose, pioglitazone, and SGLT-2 inhibitors limit insulin production, reducing the risk of hypoglycemia [23].

Polycystic ovary syndrome and insulin resistance

Recently, it has been shown that many people with PCOS have metabolic and endocrine abnormalities. The most striking of them is the presence of insulin resistance along with compensatory hyperinsulinemia. Nonetheless, the prevalence of insulin resistance and hyperinsulinemia will differ depending on how these parameters are monitored [24]. Most PCOS women exhibit metabolic syndrome symptoms such as insulin resistance, obesity, and dyslipidemia. Women of reproductive age who have polycystic ovarian syndrome and are obese have an eight times greater chance of getting type 2 diabetes [25]. PCOS also leads to insulin resistance in 50-80% of patients. Insulin resistance is fasting insulin levels of more than 20 mIU/mL and postprandial insulin levels of more than 55 mIU/mL. Insulin resistance is thought to be due to some specific genetic abnormalities. There is a post-binding abnormality with the abnormal autophosphorylation of the beta-subunit of insulin receptor and insulin substrate-1 (IRS-1). There is a decrease in tyrosine phosphorylation and an increase in serine phosphorylation, which reduces insulin metabolism. It has also been proven that the involvement of cytochrome P450c17 is seen in androgen formation, which also plays a role in abnormal insulin receptor and IRS-1 phosphorylation. It additionally induces theca cells to synthesize more androgens, resulting in dyslipidemia. Acanthosis nigricans is a clinical manifestation of insulin resistance syndrome [26]. Thus, DM is a long-term complication of PCOS. However, insulin resistance is assumed to be the etiology, leading to aberrant glucose metabolism and lipid profile, increasing the chances of developing DM and cardiovascular disease over time. Also, some studies have shown that PCOS affects the function of beta cells. In women with PCOS, insulin resistance and beta cell dysfunction increase the risk of developing DM. The risk of developing gestational diabetes is also increased [24]. However, androgens cause a slight increase in insulin resistance. Accordingly, those taking synthetic anabolic steroids and women who use oral contraceptives that include androgenic progestins may experience glucose intolerance [27]. DM is more prevalent in women with PCOS than previously thought.

As a consequence, the condition should be regarded as a risk factor for the development of diabetes. Oral glucose loading frequently reveals that women with polycystic ovarian syndrome and fasting plasma glucose levels in the 5.0-7.0 mmol/L range have reduced glucose tolerance or diabetes. As a result, the threshold for oral glucose loading should be lower in this group than in the general population [8]. Hirsutism and enlarged ovaries were the diagnostic indicators for PCOS. Additionally, it has been shown that women who meet the following conditions often experience oligomenorrhea and infertility [27]. They highly recommend that all PCOS women undergo a glucose intolerance test. This test should be performed using baseline and two-hour glucose-challenged levels rather than just evaluating the fasting glucose levels [28]. The investigations that need to be carried out to check insulin resistance are the 75 grams oral glucose tolerance test (OGTT) and the hyperinsulinemic-euglycemic glucose clamp test [29]. The lipid profile, including lipid and lipoprotein levels, should also be checked in all PCOS women with altered glucose levels.

The clinical characteristics and metabolic profiles, including the insulin sensitivity index (ISI), were compared. The findings revealed that PCOS women had significantly greater insulin responses during the OGTT, whereas their blood glucose levels were comparable to controls. Insulin resistance was higher in the PCOS participants than in the other groups. Except for age, LH, testosterone, and sex hormone-binding globulin (SHBG), there was no change in clinical features or metabolic profiles between the groups [29]. The primary treatment for insulin resistance is dietary interventions mainly composed of vegetables and unsaturated fats. A low-calorie diet can also help to reduce the ectopic fat deposition in liver and pancreas [30]. The medical treatment of choice for insulin resistance in PCOS is metformin; it has a potential long-term benefit in preventing DM. Metformin is a biguanide that improves sensitivity to insulin by increasing peripheral absorption of glucose and utilization. It helps lose weight, restores menstrual irregularity, and lowers the androgen level. It is indicated in PCOS with impaired glucose tolerance and acanthosis nigricans [31].

Lactic acidosis is the most severe adverse effect of metformin because it decreases gluconeogenesis by inhibiting pyruvate carboxylase. This enzyme transforms pyruvate to oxaloacetate, causing the enzyme to be blocked and lactic acid to accumulate. However, studies have also reported that metformin 500 mg given three times daily decreases insulin secretion and reduces the ovarian production of 17-alpha-hydroxyprogesterone [32]. Glitazones, which enhance fat deposition in fat cells and limit the degree of ectopic fat deposition, are the rational choice for improving insulin sensitivity [30]. Other helpful drugs in treating insulin resistance include GLP1 receptor agonists and empagliflozin, a sodium-glucose cotransporter-2 inhibitor [33]. However, it has been reported that anti-androgen therapy does not change insulin sensitivity in PCOS [34]. 

## Conclusions

It is now known that PCOS is usually accompanied by crucial insulin resistance and secretion defects. The much greater frequency of glucose intolerance in PCOS is explained by these abnormalities, together with obesity. In at least 50% of PCOS women, insulin resistance appears to be associated with increased serine phosphorylation of the insulin receptor. This process, which is produced by an extrinsic factor to the insulin receptor, most likely a serine/threonine kinase, is a major mechanism for human insulin resistance connected to variables impacting insulin receptor signaling. The activity of P450c17, a crucial regulator of androgen synthesis, is affected by phosphorylation of serine. PCOS has a menarchal age of onset, making it an ideal condition for researching the ontogeny of glucose metabolism anomalies and generating vast three-generation kindreds for positioning cloning studies to find type 2 DM genes.
